# Obesity and Beyond: Lifestyle Patterns and Cardiometabolic Burden in High-Risk Patients with Coronary Artery Disease—Moving Toward Personalized Prevention

**DOI:** 10.3390/diseases14020057

**Published:** 2026-02-02

**Authors:** Dariusz A. Kosior, Karol Kamiński, Zbigniew Gąsior, Marek Styczkiewicz, Aldona Kubica, Katarzyna Charkiewicz-Szeremeta, Józefa Dąbek, Piotr Michalski, Magda Łapińska, Łukasz Maciejewski, Agata Kosobucka-Ozdoba, Daniel Rabczenko, Michał H. Kosior, Piotr Jankowski

**Affiliations:** 1 Mossakowski Medical Research Institute, Polish Academy of Sciences, 02-106 Warsaw, Poland; 2 Department of Sports Medicine, Centre of Postgraduate Medical Education, 00-416 Warsaw, Poland; 3 Department of Internal Medicine and Geriatric Cardiology, Centre of Postgraduate Medical Education, 00-416 Warsaw, Poland; piotrjankowski.eu@gmail.com; 4 Department of Cardiology, Medical University of Bialystok, 15-276 Bialystok, Poland; fizklin@gmail.com; 5 Department of Population Medicine and Civilization Diseases Prevention, Medical University of Bialystok, 15-269 Białystok, Poland; magda.lapinska@umb.edu.pl; 6 II Department of Cardiology, Medical University of Silesia, 40-055 Katowice, Poland; zgasior@sum.edu.pl (Z.G.); lukasz.maciejewski1990@gmail.com (Ł.M.); 7 Department of Cardiology, John Paul II Hospital, 22-400 Zamość, Poland; styczkiewicz@interia.pl; 8 Department of Health Promotion, Collegium Medicum, Nicolaus Copernicus University, 85-067 Bydgoszcz, Poland; akubica@cm.umk.pl (A.K.); michalski.piotr@onet.eu (P.M.); a.kosobucka@cm.umk.pl (A.K.-O.); 9 Department of Cardiology and Internal Medicine, Center of Postgraduate Medical Education, Grochowski Hospital, 04-073 Warszawa, Poland; katarzyna.charkiewicz006@gmail.com; 10 Department of Cardiology, Medical University of Silesia, 40-055 Katowice, Poland; jdabek@sum.edu.pl; 11 Department of Population Health Monitoring and Analysis, National Institute of Public Health, National Institute of Hygiene—National Research Institute, 00-791 Warsaw, Poland; drabczenko@pzh.gov.pl; 12 Institute of Clinical Sciences, Maria Sklodowska-Curie Medical Academy, 03-411 Warsaw, Poland; mirolekk123@gmail.com; 13 Department of Epidemiology and Health Promotion, School of Public Health, Centre of Postgraduate Medical Education, 01-826 Warsaw, Poland

**Keywords:** obesity, secondary prevention, coronary artery disease, risk-factor control, lifestyle behaviour, personalized therapy

## Abstract

Background: Obesity substantially increases cardiovascular risk and contributes to the accumulation of cardiometabolic risk factors. Achieving optimal control of body weight and guideline-recommended targets is essential in high-risk patients, particularly in secondary prevention following acute coronary events. This study aimed to evaluate treatment strategies and lifestyle modifications undertaken by patients with obesity during long-term follow-up. Methods: This analysis included patients enrolled 6–18 months after acute coronary syndrome or coronary revascularization within the multicentre POLASPIRE II study. Standardized EUROASPIRE methodology was applied to collect clinical, anthropometric, and lifestyle-related data. Results: A total of 788 patients (mean age 65.4 ± 8.9 years; 25.8% women) were included, of whom 40.6% had obesity. No significant association between sex and BMI was observed (β = −0.48; 95% CI −1.30 to 0.31; *p* = 0.20). Increasing age was associated with lower BMI (β = −0.05; 95% CI −0.09 to −0.0001; *p* = 0.044), and higher education correlated with lower BMI (β = −1.10; 95% CI −2.00 to −0.22; *p* = 0.015). With advancing age (OR 1.02; 95% CI 1.002–1.033; *p* = 0.023) and increasing BMI (OR 1.11; 95% CI 1.076–1.138; *p* = 0.001), the number of risk factors and comorbidities increased. Higher BMI was associated with poorer control of medical risk factors (OR 1.06; 95% CI 1.03–1.10; *p* < 0.001), whereas patients with higher BMI demonstrated better control of lifestyle-related risk factors (OR 0.95; 95% CI 0.919–0.983; *p* = 0.003). Conclusions: Obesity is highly prevalent among high-risk cardiovascular patients and is associated with a greater burden of comorbidities and poorer control of medical risk factors. These findings support the need for strengthened, risk-stratified secondary prevention strategies and more personalized therapeutic approaches in patients with obesity.

## 1. Introduction

Coronary heart disease (CHD) remains one of the leading causes of morbidity and mortality worldwide and continues to impose a substantial clinical and economic burden [1]. Secondary prevention following acute coronary syndrome (ACS) is a cornerstone of contemporary cardiovascular care, aiming to reduce recurrent events and improve long-term outcomes through optimal control of modifiable risk factors [1,2].

In recent years, preventive cardiology has increasingly incorporated the principles of personalized and precision medicine, emphasizing adaptation of lifestyle, pharmacological, and behavioural interventions to individual clinical, metabolic, and psychosocial profiles [3,4,5,6,7]. Personalized approaches to cardiovascular prevention acknowledge that patient responses to standard lifestyle modifications such as dietary changes, physical activity, and smoking cessation vary considerably.

This variability reflects differences in genetic predisposition, metabolic phenotype, behavioural determinants, and the presence of comorbid conditions [4,5,6,7]. Consequently, individualized risk stratification, tailored therapeutic strategies, and patient-specific counselling have become central elements of contemporary CHD management [4,5,6,7].

This framework is particularly relevant for patients with obesity, a major and heterogeneous modifiable risk factor contributing to atherosclerosis, metabolic dysregulation, and impaired functional capacity [8,9,10,11]. Beyond body mass index (BMI), growing evidence highlights the importance of fat distribution and visceral adiposity in determining cardiometabolic risk, underscoring the limitations of BMI-based risk assessment when used in isolation [12]. In patients with established coronary artery disease, obesity is associated with earlier disease manifestation, a greater burden of comorbidities, and worse functional outcomes [8,11].

Long-term weight management in patients with CHD remains challenging in routine clinical practice. Sustained weight loss is frequently counteracted by neurohormonal and metabolic adaptations that promote weight regain, alongside substantial interindividual variability in behavioural responses to lifestyle interventions [13,14,15,16,17]. These mechanisms partly explain why improvements in lifestyle behaviours do not consistently translate into durable reductions in cardiometabolic risk [14,15,16].

Since 1994, the European Society of Cardiology (ESC) has supported the implementation and monitoring of secondary prevention guidelines through the EUROASPIRE programme, a series of cross-sectional surveys assessing preventive care practices across Europe [18,19,20,21]. These surveys have consistently demonstrated suboptimal attainment of guideline-recommended risk-factor targets and have highlighted persistent gaps in individualized counselling and therapeutic optimization [20,21]. In Poland, the POLASPIRE studies have provided detailed national-level data on secondary prevention patterns and adherence to ESC recommendations [22,23,24,25].

Despite these efforts, evidence remains limited regarding the real-world implementation of secondary prevention strategies, specifically among patients with obesity following ACS. This subgroup may derive particular benefit from precision-based preventive approaches, given its complex metabolic, behavioural, and psychosocial profile [2,7,26,27,28]. A more detailed understanding of risk-factor trajectories, lifestyle behaviours, and clinical support needs in this population is therefore essential for the development of targeted and effective interventions aligned with the principles of personalized cardiovascular medicine [4,5,6,7].

Therefore, the aim of the present study was to assess the implementation of guideline-based secondary prevention strategies in patients with obesity following ACS, with particular emphasis on personalized risk-factor modification and the role of healthcare professionals in delivering individualized lifestyle and behavioural support. By identifying existing gaps and potential areas of variability in care, this study seeks to inform the development of precision-oriented tools and interventions within contemporary cardiovascular prevention frameworks [1,7,25].

By focusing on the discordance between lifestyle-related behaviours and objective medical risk-factor control in patients with obesity, this analysis extends prior EUROASPIRE and POLASPIRE reports toward a personalized secondary prevention perspective. The novelty of this study lies in its nationwide scope and the systematic, standardized assessment of both medical and lifestyle-related risk-factor control in a real-world secondary prevention setting, enabling identification of implementation gaps relevant to personalized prevention.

## 2. Materials and Methods

### 2.1. Study Design and Framework

The POLASPIRE II study (Evaluation of the Implementation of Recommendations for Secondary Prevention of Coronary Heart Disease), conducted within the framework of the EUROASPIRE programme, was a nationwide, cross-sectional survey designed to assess the implementation of contemporary secondary prevention guidelines in Poland between 2018 and 2021 [20,21,25]. The study followed standardized EUROASPIRE methodology to ensure comparability with other participating European centres.

Six geographically diverse regions were selected to ensure national representativeness and to include centres providing the full spectrum of cardiovascular services, including interventional cardiology, cardiac surgery, acute coronary syndrome (ACS) care, and outpatient cardiology follow-up. In total, twelve cardiology departments from ten hospitals participated in the study.

### 2.2. Study Population and Eligibility Criteria

Centrally trained research teams systematically screened hospital discharge records to identify eligible patients. Eligibility criteria were as follows:

Inclusion criteria:

- Age 18–80 years at the time of index hospitalization.
- Index event: hospitalization for acute myocardial infarction or unstable angina and/or elective or emergency coronary revascularization (PCI or CABG).
- Attendance at a standardized study visit scheduled 6–18 months after the index hospitalization.
- Provision of written informed consent prior to any study procedures.

Exclusion criteria:

- Death during the index hospitalization.
- In cases of multiple qualifying hospitalizations, only the first event was considered.

All eligible participants were invited to attend the study visit. Detailed study procedures are described below.

### 2.3. Data Collection Procedures

All participating centres used standardized data collection instruments and procedures, supported by centrally coordinated quality control mechanisms. Trained study personnel conducted structured interviews, reviewed medical records, and performed standardized physical examinations. Data collection followed a predefined, standardized workflow across participating centres to ensure data quality and inter-centre comparability.

Data were collected on demographic characteristics, cardiovascular history, comorbidities, guideline-defined risk factors, lifestyle behaviours, family medical history, medication use, treatment adherence, and patient awareness of secondary prevention measures. Participants also completed validated self-report questionnaires addressing physical activity, dietary habits, and general lifestyle behaviours.

### 2.4. Anthropometric and Clinical Measurements

Anthropometric measurements were obtained in accordance with EUROASPIRE protocols [20,21]. Body weight and height were measured using calibrated scales and stadiometers, and body mass index (BMI) was calculated as weight in kilograms divided by height in metres squared (kg/m^2^). Waist circumference was measured using a non-stretch tape placed at the midaxillary line, midway between the lowest rib and the iliac crest, with participants standing in an upright position [12,20].

Blood pressure was measured on the non-dominant arm after at least five minutes of seated rest, in accordance with EUROASPIRE recommendations. Two measurements were obtained; if systolic values differed by ≥20 mmHg or diastolic values by ≥10 mmHg, a third measurement was performed. The mean of the available readings was used for statistical analyses.

### 2.5. Laboratory Assessments

Venous blood samples were collected after a 12 h overnight fast. Laboratory analyses included a complete lipid profile, fasting plasma glucose, and glycated hemoglobin (HbA1c) to assess glycemic control. All laboratory measurements were performed locally at participating centres in accredited clinical laboratories, in accordance with routine clinical standards and the EUROASPIRE methodology.

### 2.6. Smoking Status Verification

Smoking status was verified by measuring exhaled carbon monoxide concentration using a Micro+ Smokerlyzer^®^ device (Bedfont Scientific, Maidstone, UK), in accordance with established EUROASPIRE procedures [20]. Active smoking was defined as continued smoking at the time of the study visit among participants who reported smoking during the month preceding the index hospitalization, in accordance with EUROASPIRE definitions.

### 2.7. Definitions of Risk Factor Control

Risk factor control was assessed according to contemporary international clinical guidelines, primarily those issued by the European Society of Cardiology [1]. Blood pressure control was defined as systolic/diastolic values <130/80 mmHg in patients younger than 70 years and <140/80 mmHg in those aged 70 years or older. Adequate lipid control was defined as achieving a low-density lipoprotein cholesterol (LDL-C) concentration <70 mg/dL. As recommendations propose lower LDL-C targets (<55 mg/dL) for very high-risk patients, applying contemporary thresholds would likely further accentuate the observed gaps in secondary prevention. Glycemic control was considered appropriate when HbA1c was <7.0% [28].

Abdominal obesity was defined using sex-specific thresholds: waist circumference ≥94 cm in men and ≥80 cm in women. Physical activity was assessed using the standardized and validated EUROASPIRE physical activity questionnaire. Weight control was defined as any intentional attempt to reduce or maintain body weight during the three months preceding the study visit, irrespective of the method used [26].

For analytical purposes, two categories of modifiable cardiovascular risk factors were defined. Medical risk factors included biochemical and clinical parameters (LDL-C, fasting plasma glucose, HbA1c, and blood pressure). Lifestyle-related risk factors comprised behavioural determinants, including cigarette smoking, physical activity, and weight management efforts. This classification aligns with the personalized prevention framework.

### 2.8. Ethical Considerations

The study was conducted in accordance with the Declaration of Helsinki, the principles of Good Clinical Practice (GCP), and applicable national regulations governing biomedical research in Poland. Ethical approval was obtained from the Bioethics Committee of the Centre of Postgraduate Medical Education (CMKP), Warsaw, Poland (Approval No. 129/2022, dated 12 October 2022).

All participants were informed about the study objectives, procedures, potential risks, and benefits prior to enrolment. Written informed consent was obtained from each participant before any study-related procedures were performed. All data were pseudonymized and processed in accordance with the EU General Data Protection Regulation (GDPR; Regulation (EU) 2016/679).

### 2.9. Statistical Analysis

A complete-case analysis was performed, restricting statistical evaluations to participants with non-missing BMI and demographic data. Categorical variables are presented as frequencies and percentages, and continuous variables as means with standard deviations.

Comparisons between BMI categories (normal weight/overweight vs. obesity) were conducted using chi-square or Fisher’s exact tests for categorical variables and the Mann–Whitney U test for continuous variables. Associations between BMI and the number of medical or lifestyle-related risk factors and comorbidities were assessed using ordered logistic regression models adjusted for age and sex, selected a priori. Ordinary least-squares linear regression was used to examine the association between demographic characteristics and BMI. All statistical tests were two-tailed, with a *p* value < 0.05 considered statistically significant. Analyses were performed using STATA software (StataCorp LLC, College Station, TX, USA; Release 18, 2023).

## 3. Results

### 3.1. Study Population Characteristics

Of the 801 individuals enrolled in the POLASPIRE II study, complete data were available for 788 participants, who were included in the final analysis. The mean age of the cohort was 65.4 ± 8.9 years, and women accounted for 25.8% of the study population. At the time of the study visit, 19.1% of participants were current smokers. Hypertension was present in 77.7% of patients, diabetes mellitus in 35.3%, and hypercholesterolaemia in 79.3%.

### 3.2. Anthropometric Characteristics

**Figure 1:**
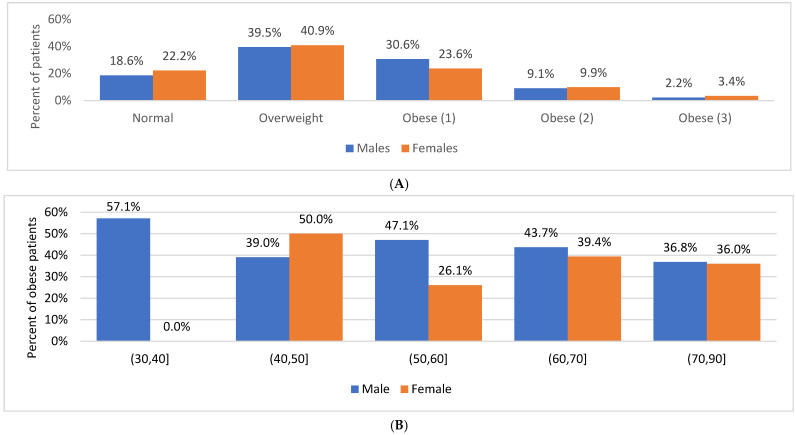
Distribution of body mass index in study group (**A**) and by age at the time of the interview (**B**).

The mean body mass index (BMI) was 29.2 ± 4.8 kg/m^2^. Obesity (BMI ≥ 30 kg/m^2^) was identified in 40.6% of patients, while 39.8% were classified as overweight. Central obesity was significantly more prevalent among women than men (71.9% vs. 48.4%; *p* < 0.001). Morbid obesity (BMI ≥ 40 kg/m^2^) was observed in 2.5% of the cohort, whereas 19.5% of patients had a normal BMI. No significant sex-related differences in obesity prevalence were observed (Table 1 and Table 2). Figure 1 illustrates the distribution of body mass index in the study population and across age groups. Statistical comparisons between BMI categories are presented in Table 1 and Table 2.

### 3.3. Clinical Risk Profile According to BMI

Patients with obesity exhibited a higher prevalence of diabetes mellitus (47.1% vs. 27.0%; *p* = 0.001), hypertension (84.8% vs. 72.9%; *p* = 0.001), and earlier clinical manifestation of coronary heart disease (20.4% vs. 14.0%; *p* = 0.025) compared with patients without obesity. Obesity was also associated with lower educational attainment (*p* = 0.002) and professional inactivity related to retirement or disability (*p* = 0.034).

Individuals with obesity tended to report lower levels of physical activity and were more frequently current or former smokers. Knowledge of guideline-recommended therapeutic targets varied across risk factors; while 87.8% of patients were aware of recommended blood pressure values, only 43.3% reported knowledge of target fasting glucose levels, and 28.3% were aware of recommended cholesterol targets.

### 3.4. Risk Factor Control

At a follow-up assessment, an LDL-C concentration <70 mg/dL was achieved by 43.5% of the overall cohort, and an HbA1c level <7.0% by 83.9%. Poor glycemic control (HbA1c ≥ 7.0%) was more frequent among patients with obesity compared with those with normal weight or overweight (21.1% vs. 12.8%; *p* = 0.004). In contrast, achievement of LDL-C <70 mg/dL did not differ significantly between patients without obesity and those with obesity (45.6% vs. 40.4%; *p* = 0.19) (Table 3).

Although 92.7% of participants reported regular blood pressure monitoring, optimal blood pressure control was achieved by 83.9% of the cohort; however, control rates were significantly lower among patients with obesity compared with those without obesity (78.9% vs. 87.2%; *p* = 0.003).

Overall, optimal control of all medical risk factors was achieved by 13.1% of patients, including only 7.7% of individuals with obesity. Conversely, 6.1% of the cohort did not meet any guideline-recommended medical targets, a proportion that was higher among patients with obesity (8.5%; *p* = 0.002).

### 3.5. Lifestyle Behaviours

Among patients with obesity, 19.7% reported that they had not been informed by a healthcare professional about their obesity since the index hospitalization, and 35.7% reported not receiving dietary recommendations. Among those who received counselling, 57.6% reported adherence to dietary advice.

Following hospitalization, 56.7% of patients with obesity attempted to lose weight, whereas 43.3% reported efforts to maintain their weight. Approximately half of obese individuals declared no intention to pursue weight reduction within the subsequent six months. Pharmacological weight-reducing therapy was recommended in 4.2% of patients with obesity.

Participation in a cardiac prevention and rehabilitation programme was reported by 74.1% of the overall cohort and did not differ significantly across BMI categories (*p* = 0.60).

Regular daily physical activity was reported by 25.9% of patients overall and by 21.0% of those with obesity. Women with obesity were significantly less likely to engage in regular physical activity than men (16.0% vs. 29.9%). Complete physical inactivity was more common among obese patients (19.3%) and was particularly prevalent among obese women (29.7% vs. 16.1%; *p* = 0.002).

The achievement of guideline-recommended physical activity levels (≥3–5 sessions per week, 20–30 min per session) was reported by 20.4% of all participants and only by 14.2% of patients with obesity, with markedly lower rates among obese women compared with men (4.1% vs. 17.4%) (Table 4).

Across the entire cohort, optimal control of all lifestyle-related cardiovascular risk factors was achieved by 11.2% of patients; among individuals with obesity, this proportion was 13.1%.

### 3.6. Smoking Patterns

At the time of the study visit, 19.1% of participants were current smokers, with no significant differences across BMI categories. Prior to hospitalization, 42.0% of the cohort were active smokers, 21.0% quit smoking immediately after discharge, and an additional 10.0% quit at a later time point.

Among persistent smokers, 41.3% reported no intention to quit despite awareness of smoking-related health risks. Nicotine replacement therapy was used by 4.5% of the study population.

### 3.7. Regression Analyses

Sex was not significantly associated with optimal control of lifestyle-related (OR 0.885; 95% CI 0.598–1.309; *p* = 0.541) or medical (OR 0.963; 95% CI 0.683–1.358; *p* = 0.830) risk factors. Increasing age was associated with better control of both medical (OR 0.977; 95% CI 0.961–0.993; *p* = 0.006) and lifestyle-related (OR 0.962; 95% CI 0.945–0.980; *p* < 0.001) risk factors.

Higher BMI was associated with poorer control of medical risk factors (OR 1.064; 95% CI 1.031–1.098; *p* < 0.001) but with better control of lifestyle-related risk factors (OR 0.951; 95% CI 0.919–0.983; *p* = 0.003).

Both increasing age (OR 1.018; 95% CI 1.002–1.033; *p* = 0.023) and BMI (OR 1.106; 95% CI 1.076–1.138; *p* = 0.001) were independently associated with a higher number of comorbidities and cardiometabolic risk factors, whereas sex was not (OR 1.099; 95% CI 0.810–1.491; *p* = 0.543).

**Figure 2:**
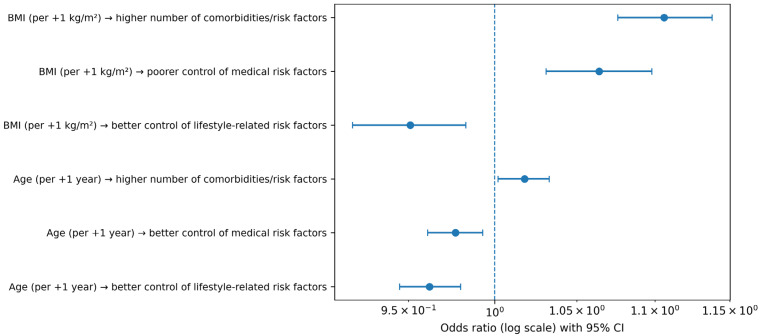
Forest plot summarizing selected multivariable associations (odds ratios with 95% confidence intervals).

Sex was not significantly associated with BMI (β = −0.48; 95% CI −1.30 to 0.31; *p* = 0.200). Increasing age was associated with a modest but statistically significant decrease in BMI (β = −0.05; 95% CI −0.09 to −0.0001; *p* = 0.044). Higher educational attainment was associated with lower BMI, including secondary education (β = −1.10; 95% CI −2.00 to −0.22; *p* = 0.015) and higher education (β = −1.80; 95% CI −2.80 to −0.73; *p* < 0.001). Employment status was not significantly associated with BMI, although unemployed individuals tended to have higher values (β = 3.3; 95% CI −0.10 to 6.6; *p* = 0.057) (Table 5). Key multivariable associations derived from regression analyses are summarized in Figure 2 and detailed in Table 5.

## 4. Discussion

This nationwide analysis highlights substantial opportunities to reduce cardiovascular risk among high-risk patients with established coronary artery disease (CAD) in Poland through more individualized secondary prevention strategies. Our findings confirm the persistently high prevalence of overweight and obesity following acute coronary syndrome (ACS) or coronary revascularization, reinforcing excess body weight as a major barrier to effective risk reduction [1,2,28]. The added value of this study lies in its nationwide scope and the comprehensive, standardized assessment of both medical and lifestyle-related risk factors in a real-world secondary prevention setting.

Body mass index (BMI) was not associated with sex, whereas increasing age and higher educational attainment were linked to lower BMI values. Although employment status was not significantly associated with BMI, non-working individuals tended to exhibit higher values. These patterns are consistent with earlier national and European observations and underscore heterogeneity across patient subgroups, reinforcing the need for tailored, patient-centred preventive approaches [20,21,22,28].

In this cohort, obesity was associated with a greater burden of comorbidities and poorer control of medical risk factors. Notably, lifestyle-related behaviours, including physical activity and smoking cessation, were more frequently reported as controlled among individuals with higher BMI. This observation is biologically plausible in the context of well-described counter-regulatory metabolic and neurohormonal adaptations that limit sustained weight reduction and may attenuate downstream cardiometabolic improvements despite behavioural efforts [13,14,15,16,17]. These findings highlight a clinically relevant mismatch between patients’ lifestyle engagement and objective cardiometabolic target attainment, suggesting that behavioural effort alone may be insufficient to overcome obesity-related biological resistance.

Obesity is a heterogeneous condition in which BMI alone does not fully capture fat distribution, metabolic phenotype, or cardiometabolic risk, supporting the interpretation that reported lifestyle engagement does not necessarily translate into measurable improvements in biological targets [8,10,11,12].

These findings highlight the heterogeneity of patient responses and emphasize the importance of structured, personalized behavioural support and realistic, patient-specific goal setting [3,4,5,6,7].

Importantly, inadequate control of medical risk factors in patients with obesity appears to reflect not only individual behaviours but also systemic determinants, including healthcare organization, treatment intensity, and physician practice patterns. From a health-system perspective, these findings support the need for structured, multidisciplinary secondary prevention pathways that integrate pharmacological optimization with personalized lifestyle and behavioural support.

Despite high self-reported rates of blood pressure monitoring, optimal hypertension control remained low, consistent with Polish population data documenting limited patient knowledge and imperfect implementation of hypertension criteria and related complications [29]. This observation is concordant with registry data indicating persistent gaps in guideline implementation and adherence to lifestyle recommendations across Europe [18,19,20,21,26].

Six to eighteen months after ACS or coronary revascularization, the implementation of evidence-based secondary prevention recommendations remained suboptimal. Persistently high prevalences of obesity and smoking are consistent with earlier national reports and global observations from EUROASPIRE, INTERASPIRE, and PURE, indicating limited progress over time [20,30].

Beyond biological and behavioural contributors, residual cardiovascular risk after ACS is increasingly understood as multifactorial, involving both suboptimal target attainment and non-traditional drivers, which supports intensified, individualized post-ACS management strategies [2,31].

The present results are also concordant with multinational European data. EUROASPIRE surveys have repeatedly documented high rates of obesity, smoking, physical inactivity, and insufficient control of hypertension, dyslipidemia, and diabetes among patients with CAD [18,19,20,21]. INTERASPIRE further highlighted substantial international and regional variation in secondary prevention performance, supporting the need for system-level and patient-level solutions rather than uniform, one-size-fits-all approaches [32]. Comparisons with CLARIFY suggest that Polish patients with stable CAD face challenges comparable to those observed in other European populations in achieving guideline-recommended targets [31,33]. Collectively, these observations support a shift towards more individualized, patient-centred approaches embedded in guideline frameworks for both chronic coronary syndromes and prevention after acute events [1,34].

Beyond biomedical risk factors, sociodemographic and psychological determinants substantially influence lifestyle adherence and long-term outcomes. Educational attainment, economic resources, and mental health affect treatment engagement and behavioural maintenance in patients with CAD [35,36,37]. Although mental health variables were not directly assessed, available POLASPIRE II data indicate a relevant burden of anxiety and depression, supporting their potential role in shaping physical inactivity, smoking, and obesity-related behaviours [38].

Taken together, these findings support a shift from uniform, target-driven secondary prevention towards more individualized, patient-centred strategies that integrate biological profiling, behavioural assessment, and psychosocial context. Such an approach aligns with contemporary ESC guideline frameworks and may help reduce residual cardiovascular risk in high-risk patients with coronary artery disease [1,3,7,34].

In parallel, contemporary evidence suggests that BMI trajectories carry long-term prognostic implications, supporting longitudinal, individualized risk communication and sustained follow-up beyond isolated post-ACS visits [39].

### Implications for Personalized Medicine

The present findings highlight a persistent mismatch between patients’ self-reported lifestyle efforts and objective control of medical risk factors, suggesting that conventional secondary prevention strategies may insufficiently address the heterogeneity of patients recovering from ACS [1,2,3,20,25]. This discrepancy supports the need for individualized approaches that extend beyond uniform risk-factor targets and incorporate multidimensional risk profiling [4,5,6,7,36].

A comprehensive personalized prevention model should account for biological variability, including differences in metabolic status, body composition, inflammatory burden, and genetic predisposition, as these factors influence both disease progression and therapeutic response [5,6,7,10,11,12,34]. Behavioural characteristics such as motivation, readiness for change, and perceived barriers are critical determinants of lifestyle adherence, while psychological factors modulate health behaviours and engagement with treatment [32,33,36]. Together, these dimensions support integrating individualized counselling, tailored exercise prescriptions, adaptive nutritional strategies, and precision-directed pharmacotherapy to improve implementation of guideline-based secondary prevention [1,2,3,6,7,39].

Digital health technologies may serve as valuable adjuncts to personalized prevention. Remote monitoring, app-based coaching, and individualized feedback systems have the potential to enhance adherence and support long-term self-management, particularly in patients facing sustained behavioural challenges and relapse-prone weight trajectories [14,15,16,17,36]. Integration of such tools into routine care may help bridge existing gaps and align secondary prevention more closely with precision prevention principles [7,37].

## 5. Strengths and Limitations

**Strengths.** This study analyzed a large, well-characterized cohort reflecting real-world secondary prevention among Polish patients with CAD assessed 6–18 months after an acute coronary event. The use of standardized data collection procedures aligned with established EUROASPIRE methodology supports comparability with other European datasets [18,19,20,21]. The multidimensional approach, integrating clinical, behavioural, and sociodemographic variables, enabled a comprehensive assessment of gaps in secondary prevention and aligns with broader precision prevention frameworks [7,36,38].

**Limitations.** Several limitations of the present study should be acknowledged. The present study did not include a systematic quantitative assessment of coronary imaging data, such as coronary computed tomography angiography or intravascular ultrasound-derived parameters. Imaging procedures were performed according to routine clinical indications to establish the diagnosis of coronary artery disease and to guide revascularisation, rather than as part of a predefined research protocol. Consequently, formal correlations between imaging characteristics and lifestyle-related parameters could not be assessed. This limitation reflects real-world clinical practice in large nationwide cohorts and should be considered when interpreting the findings. Future studies integrating standardized coronary imaging with lifestyle and metabolic profiling may provide further insights into the relationship between obesity, behavioural factors, and coronary atherosclerosis.

The study included only patients who consented to participate after ACS or revascularization, which may limit generalizability. Individuals aged ≥80 years were excluded. Lifestyle behaviours were self-reported and therefore subject to recall and social desirability bias. BMI does not capture body composition or fat distribution, and measures such as waist circumference may better reflect visceral adiposity-related risk [11,12]. Mental health variables were not directly assessed, limiting the interpretation of psychosocial drivers of behaviour. Although the analysis was conducted within a single country, the findings are consistent with European and global trends reported in EUROASPIRE, INTERASPIRE, and PURE [20,30,38].

## 6. Conclusions

This nationwide evaluation of patients following ACS or coronary revascularization in Poland reveals persistent deficiencies in contemporary secondary cardiovascular prevention. Overweight and obesity remain highly prevalent and are associated with a greater burden of cardiometabolic risk factors and suboptimal control of blood pressure, lipid levels, and glycemia [1,8,10,11,12,28]. Despite reported engagement in selected lifestyle behaviours, achievement of guideline-recommended therapeutic targets remains limited, consistent with broader European and global registry data [20,30,38].

These findings support the need for more effective, personalized prevention strategies that integrate biological profiling, behavioural assessment, psychological support, and sociodemographic context [5,6,7,32,33,34,35,36,37]. Tailored, patient-centred interventions supported by structured follow-up, guideline-based intensification of therapy, and digital health solutions may enhance adherence, improve risk-factor control, and ultimately reduce residual cardiovascular risk in high-risk CAD populations [1,2,3,7,37,39,40].

## Figures and Tables

**Table 1:** Characteristics of the study population.

	Obesity Status			Obesity Group			
	Total (*n* = 788)	Not Obese (*n* = 468)	Obese (*n* = 320)	Significance ^1^	Obese (*n* = 320)		Significance ^2^
					Men (*n* = 245)	Women (*n* = 75)	
**age (years)**	65.4 ± 8.9	65.6 ± 9.1	65.0 ± 8.7	0.231	64.2 ± 8.8	67.9 ± 7.8	0.002
**sex:**							
male	585 (74.2%)	340 (72.6%)	245 (76.6%)	0.217			
female	203 (25.8%)	128 (27.4%)	75 (23.4%)				
**medical history prior to hospitalization:**							
myocardial infarction	192 (24.6%)	111 (24.0%)	81 (25.6%)	0.598	66 (27.4%)	15 (20.0%)	0.201
CABG	47 (6.0%)	27 (5.8%)	20 (6.3%)	0.774	17 (7.0%)	3 (4.0%)	0.428
PCI	256 (32.7%)	150 (32.2%)	106 (33.3%)	0.737	81 (33.3%)	25 (33.3%)	0.999
unstable angina	55 (7.1%)	29 (6.3%)	26 (8.2%)	0.305	18 (7.4%)	8 (10.8%)	0.355
stroke	43 (5.5%)	22 (4.7%)	21 (6.6%)	0.253	35 (14.5%)	5 (6.7%)	0.076
peripheral artery disease	35 (4.5%)	21 (4.5%)	14 (4.4%)	0.947	11 (4.5%)	3 (4.0%)	0.999
congestive heart failure	49 (6.3%)	26 (5.6%)	23 (7.3%)	0.342	20 (8.3%)	3 (4.1%)	0.220
hypertension	606 (77.7%)	339 (72.9%)	267 (84.8%)	0.001	201 (83.8%)	66 (88.0%)	0.371
diabetes	267 (35.3%)	120 (27.0%)	147 (47.1%)	0.001	110 (46.2%)	37 (50.0%)	0.569
glucose intolerance	47 (7.3%)	25 (6.2%)	22 (9.0%)	0.192	18 (9.7%)	4 (6.7%)	0.471
hypercholesterolemia	606 (79.3%)	355 (78.4%)	251 (80.7%)	0.433	188 (79.3%)	63 (85.1%)	0.269
chronic obstructive pulmonary disease	32 (4.6%)	14 (3.4%)	18 (6.4%)	0.069			
history of kidney disease	107 (13.7%)	65 (13.9%)	42 (13.4%)	0.820	30 (12.5%)	12 (16.2%)	0.412
premature CAD ^3,4^	114 (16.6%)	57 (14.0%)	57 (20.4%)	0.025	47 (22.5%)	10 (14.3%)	0.141
**medication:**							
antiplatelets	717 (91.5%)	432 (92.7%)	285 (89.6%)	0.130	215 (88.1%)	70 (94.6%)	0.109
blood pressure-lowering drugs	767 (97.8%)	454 (97.4%)	313 (98.4%)	0.344	240 (98.4%)	73 (98.6%)	0.999
lipid-lowering drugs	732 (93.4%)	431 (92.5%)	301 (94.7%)	0.232	232 (95.1%)	69 (93.2%)	0.558
hypoglycemic drugs	282 (36.0%)	135 (29.0%)	147 (46.2%)	0.001	113 (46.3%)	34 (45.9%)	0.956
**specialization of the physician:**							
general practitioner	542 (68.8%)	319 (68.2%)	223 (69.7%)	0.650	167 (68.2%)	56 (74.7%)	0.284
cardiologist	698 (88.6%)	424 (90.6%)	274 (85.6%)	0.031	206 (84.1%)	68 (90.7%)	0.155
diabetologist/endocrinologist	111 (14.1%)	51 (10.9%)	60 (18.8%)	0.002			
other	100 (12.7%)	55 (11.8%)	45 (14.1%)	0.339	2 (0.8%)	0 (0.0%)	0.999
no regular checkups	4 (0.5%)	2 (0.4%)	2 (0.6%)	0.999	2 (0.8%)	0 (0.0%)	0.999
**educational status:**				0.002			0.476
primary education	145 (18.4%)	72 (15.4%)	73 (22.9%)		55 (22.5%)	18 (24.0%)	
secondary education	431 (54.8%)	252 (53.8%)	179 (56.1%)		134 (54.9%)	45 (60.0%)	
higher education	211 (26.8%)	144 (30.8%)	67 (21.0%)		55 (22.5%)	12 (16.0%)	
**professional status**				0.034			0.001
unemployed	363 (46.1%)	225 (48.1%)	138 (43.3%)		121 (49.6%)	17 (22.7%)	
employed	8 (1.0%)	1 (0.2%)	7 (2.2%)		4 (1.6%)	3 (4.0%)	
housewife	9 (1.1%)	5 (1.1%)	4 (1.3%)		0 (0.0%)	4 (5.3%)	
retired/pensioner	407 (51.7%)	237 (50.6%)	170 (53.3%)		119 (48.8%)	51 (68.0%)	

^1^ Wilcoxon rank sum test; Pearson’s Chi-squared test; Fisher’s exact test. ^2^ Wilcoxon rank sum test; Fisher’s exact test; Pearson’s Chi-squared test. ^3^ Premature CAD defined as *occurrence of first clinical manifestation in women less than 60 years and men less 55 years of age*. ^4^ As reported by the patients at the time of the study visit. Significance 1 refers to comparisons between patients with obesity and those without obesity, whereas significance 2 refers to sex-specific comparisons within the obese group. Abbreviations: CABG—coronary artery bypass graft; PCI—percutaneous coronary intervention; CAD—coronary artery disease.

**Table 2:** Anthropometric data, patient’s lifestyle and undertaken or planned interventions regarding body weight (reported by the patients at the time of the study visit).

	Obesity Status				Obesity Group		
	Total (*n* = 788)	Not Obese (*n* = 468)	Obese (*n* = 320)	Significance ^1^	Obese (*n* = 320)		Significance ^2^
					Men (*n* = 245)	Women (*n* = 75)	
**BMI [kg/m^2^]**	29.2 ± 4.8	26.1 ± 2.6	33.8 ± 3.3	0.001	33.6 (3.2)	34.4 (3.8)	0.225
17.0–24.9 kg/m^2^	154 (19.5%)	154 (32.9%)	0 (0.0%)	0.001	0 (0.0%)	0 (0.0%)	0.219
25.0–29.9 kg/m^2^	314 (39.8%)	314 (67.1%)	0 (0.0%)		0 (0.0%)	0 (0.0%)	
30.0–34.9 kg/m2 obesity type 1	227 (28.8%)	0 (0.0%)	227 (70.9%)		179 (73.1%)	48 (64.0%)	
35.0–39.9 kg/m2 obesity type 2	73 (9.3%)	0 (0.0%)	73 (22.8%)		53 (21.6%)	20 (26.7%)	
40.0 kg/m2 and more obesity type 3	20 (2.5%)	0 (0.0%)	20 (6.3%)		13 (5.3%)	7 (9.3%)	
**waist circumference**							
<94 cm in men; <80 cm in women	366 (46.4%)	327 (69.8%)	39 (12.2%)	0.001	38 (15.5%)	1 (1.3%)	0.002
ever been informed about the incorrect body weight	380 (48.3%)	124 (26.5%)	256 (80.3%)	0.001	190 (77.9%)	66 (88.0%)	0.054
ever been told they have unhealthy diet	403 (51.2%)	198 (42.3%)	205 (64.3%)	0.001	158 (64.8%)	47 (62.7%)	0.741
tried to actively lose weight in last month 6 months	274 (34.8%)	93 (19.9%)	181 (56.7%)	0.001	141 (57.8%)	40 (53.3%)	0.496
tried to maintain body weight (not gain weight) in last month	431 (54.8%)	222 (47.4%)	209 (65.5%)	0.001	158 (64.8%)	51 (68.0%)	0.605
seriously consider achieving their desired body weight within the next 6 months	262 (33.3%)	100 (21.4%)	162 (50.8%)	0.001	130 (53.3%)	32 (42.7%)	0.108
follow dietary recommendation	363 (51.3%)	200 (47.2%)	163 (57.6%)	0.007	118 (55.1%)	45 (65.2%)	0.141
participate in regular physical activity	250 (35.4%)	156 (36.8%)	94 (33.2%)	0.330	77 (36.0%)	17 (24.6%)	0.082
use weight-reducing drugs	28 (4.0%)	16 (3.8%)	12 (4.2%)	0.755	5 (2.3%)	7 (10.1%)	0.011
**dietary habits:**							
salt intake reduction	433 (61.2%)	266 (62.7%)	167 (59.0%)	0.319	125 (58.4%)	42 (60.9%)	0.718
fat intake reduction	491 (69.7%)	296 (69.8%)	195 (69.6%)	0.962	145 (68.4%)	50 (73.5%)	0.423
calorie intake reduction	421 (60.1%)	240 (57.0%)	181 (64.6%)	0.043	139 (65.6%)	42 (61.8%)	0.568
vegetable and fruit intake increase	503 (71.0%)	298 (70.1%)	205 (72.4%)	0.505	153 (71.5%)	52 (75.4%)	0.532
fish intake increase	398 (56.4%)	246 (58.2%)	152 (53.7%)	0.243	121 (56.5%)	31 (44.9%)	0.092
fatty fish intake increase	288 (41.2%)	179 (42.4%)	109 (39.4%)	0.420	87 (41.4%)	22 (32.8%)	0.210
carbohydrates intake reduction	435 (61.5%)	250 (59.0%)	185 (65.4%)	0.086	137 (64.0%)	48 (69.6%)	0.400
alcohol intake reduction	298 (42.9%)	178 (42.5%)	120 (43.5%)	0.795	97 (46.4%)	23 (34.3%)	0.083
rehabilitation programme participation within 3 months following the indexed hospitalization	340 (74.1%)	212 (74.9%)	128 (72.7%)	0.604	101 (72.1%)	27 (75.0%)	0.731

^1^ Wilcoxon rank sum test; Pearson’s Chi-squared test. ^2^ Wilcoxon rank sum test; Fisher’s exact test; Pearson’s Chi-squared test. Significance 1 refers to comparisons between patients with obesity and those without obesity, whereas significance 2 refers to sex-specific comparisons within the obese group. Abbreviations: BMI—body mass index.

**Table 3:** Cardiovascular risk factor control.

	Obesity Status				Obesity Group		
	Total (*n* = 788)	Not Obese (*n* = 468)	Obese (*n* = 320)	Significance ^1^	Obese (*n* = 320)		Significance ^2^
					Men *n* = 245	Women *n* = 75	
**no. of patients who were measured since hospital discharge:**							
waist circumference	257 (33.2%)	157 (34.2%)	100 (31.7%)	0.475	79 (32.9%)	44 (59.5%)	0.386
body weight	480 (61.9%)	280 (60.7%)	200 (63.7%)	0.405	156 (65.0%)	21 (28.0%)	0.425
blood pressure	724 (92.7%)	428 (92.2%)	296 (93.4%)	0.550	227 (93.8%)	69 (92.0%)	0.598
cholesterol level	579 (77.9%)	343 (77.8%)	236 (78.1%)	0.905	182 (79.5%)	54 (74.0%)	0.322
glucose level	574 (76.6%)	338 (75.4%)	236 (78.4%)	0.348	179 (78.2%)	57 (79.2%)	0.857
hemoglobin A1c	189 (28.7%)	100 (25.3%)	89 (33.8%)	0.018	65 (33.5%)	24 (34.8%)	0.847
**no. of patients who were aware of the values of their parameters:**							
waist circumference	380 (50.2%)	225 (50.3%)	155 (50.0%)	0.928	124 (52.3%)	31 (42.5%)	0.141
body weight	692 (88.8%)	404 (87.1%)	288 (91.4%)	0.058	223 (91.8%)	65 (90.3%)	0.691
blood pressure	676 (87.8%)	395 (86.2%)	281 (90.1%)	0.112	217 (90.4%)	64 (88.9%)	0.704
cholesterol level	189 (28.3%)	122 (30.7%)	67 (24.7%)	0.091	48 (22.6%)	19 (32.2%)	0.132
glucose level	391 (56.7%)	237 (57.7%)	237 (57.7%)	0.555	90 (41.5%)	34 (55.7%)	0.048
hemoglobin A1c	54 (8.1%)	26 (6.6%)	28 (10.4%)	0.073	20 (9.6%)	8 (13.3%)	0.407
**medical risk factors:**							
LDL < 70 mg/dL	285 (43.5%)	180 (45.6%)	105 (40.4%)	0.190	82 (41.2%)	23 (37.7%)	0.626
blood pressure optimal control	558 (83.9%)	348 (87.2%)	210 (78.9%)	0.003	67 (27.6%)	22 (30.1%)	0.669
hemoglobin A1c < 7%	558 (83.9%)	348 (87.2%)	210 (78.9%)	0.004	166 (80.6%)	44 (73.3%)	0.225
**total no. of well-controlled medical risk factors:**							
0	38 (6.1%)	17 (4.5%)	21 (8.5%)	0.002	12 (6.3%)	9 (15.8%)	0.168
1	256 (40.9%)	145 (38.4%)	111 (44.8%)		89 (46.6%)	22 (38.6%)	
2	250 (39.9%)	153 (40.5%)	97 (39.1%)		75 (39.3%)	22 (38.6%)	
3	82 (13.1%)	63 (16.7%)	19 (7.7%)		15 (7.9%)	4 (7.0%)	
**lifestyle risk factors:**							
intense physical activity	270 (34.3%)	174 (37.2%)	96 (30.1%)	0.040	79 (32.4%)	17 (22.7%)	0.109
non-smoking	150 (19.1%)	88 (18.8%)	62 (19.4%)	0.825	55 (22.5%)	7 (9.3%)	0.011
active weight reduction within last 30 days	274 (34.8%)	93 (19.9%)	181 (56.7%)	0.001	141 (57.8%)	40 (53.3%)	0.496
active weight control within last 30 days	431 (54.8%)	222 (47.4%)	209 (65.5%)	0.001	158 (64.8%)	51 (68.0%)	0.605
active weight control or reduction within last 30 days *	473 (60.1%)	234 (50.0%)	239 (74.9%)	0.001	179 (73.4%)	60 (80.0%)	0.246
**total no. of well-controlled lifestyle risk factors:**							
0	48 (9.0%)	35 (11.7%)	13 (5.5%)	0.004	10 (5.1%)	3 (7.5%)	0.280
1	159 (29.7%)	97 (32.3%)	62 (26.3%)		56 (28.6%)	6 (15.0%)	
2	222 (41.4%)	106 (35.3%)	116 (49.2%)		93 (47.4%)	23 (57.5%)	
3	107 (20.0%)	62 (20.7%)	45 (19.1%)		37 (18.9%)	8 (20.0%)	

* Parameter used in the analysis as a lifestyle risk factor.^1^ Wilcoxon rank sum test; Pearson’s Chi-squared test. ^2^ Wilcoxon rank sum test; Fisher’s exact test; Pearson’s Chi-squared test. Significance 1 refers to comparisons between patients with obesity and those without obesity, whereas significance 2 refers to sex-specific comparisons within the obese group.

**Table 4:** Lifestyle and habits related to smoking and physical activity (reported by the patients at the time of the study visit).

	Obesity Status				Obesity Group		
	Total (*n* = 788)	Not Obese (*n* = 468)	Obese (*n* = 320)	Significance ^1^	Obese (*n* = 320)		Significance ^2^
					Men (*n* = 245)	Women (*n* = 75)	
**cigarette smoking**				0.010			0.001
never smoked	251 (31.9%)	168 (35.9%)	83 (26.0%)		48 (19.7%)	35 (46.7%)	
smoked but quit	386 (49.0%)	212 (45.3%)	174 (54.5%)		141 (57.8%)	33 (44.0%)	
current smoker	150 (19.1%)	88 (18.8%)	62 (19.4%)		55 (22.5%)	7 (9.3%)	
nicotine replacement therapy	32 (4.5%)	15 (3.5%)	17 (6.0%)	0.123	13 (6.0%)	4 (5.8%)	0.999
**physical activity**				0.001			0.002
no physical activity	123 (15.8%)	62 (13.4%)	61 (19.3%)		39 (16.1%)	22 (29.7%)	
light physical activity in the last week	415 (53.2%)	232 (50.0%)	183 (57.9%)		137 (56.6%)	46 (62.2%)	
moderate physical activity (1–2× 20–30 min a week)	83 (10.6%)	56 (12.1%)	27 (8.5%)		24 (9.9%)	3 (4.1%)	
high physical activity (3–5× 20–30 min a week)	159 (20.4%)	114 (24.6%)	45 (14.2%)		42 (17.4%)	3 (4.1%)	
**do you exercise regularly?**				0.062			0.001
yes, for over 6 months	204 (25.9%)	136 (29.1%)	68 (21.3%)		63 (25.8%)	5 (6.7%)	
yes, for less than 6 months	45 (5.7%)	28 (6.0%)	17 (5.3%)		10 (4.1%)	7 (9.3%)	
no, but I plan to start within the next 30 days	58 (7.4%)	31 (6.6%)	27 (8.5%)		25 (10.2%)	2 (2.7%)	
no, but I plan to start within the next 6 months	77 (9.8%)	46 (9.8%)	31 (9.7%)		21 (8.6%)	10 (13.3%)	
no, and I don’t plan to start	300 (38.1%)	177 (37.8%)	123 (38.6%)				
**attending a sports class or club**	78 (11.0%)	51 (12.1%)	27 (9.5%)	0.296	23 (10.7%)	4 (5.8%)	0.224

^1^ Wilcoxon rank sum test; Pearson’s Chi-squared test. ^2^ Wilcoxon rank sum test; Fisher’s exact test; Pearson’s Chi-squared test. Significance 1 refers to comparisons between patients with obesity and those without obesity, whereas significance 2 refers to sex-specific comparisons within the obese group.

**Table 5:** Impact of demographic factors on BMI level.

		Beta	95% C.I.	*p*-Value
**sex**	male vs. female	−0.48	−1.3, 0.31	0.200
**age**	[1 year increase]	−0.05	−0.09, 0.0001	0.044
**education level**	secondary vs. below secondary	−1.10	−2.0, −0.22	0.015
	above secondary vs. below secondary	−1.80	−2.8, −0.73	0.001
**employment**	unemployed vs. employed	3.30	−0.10, 6.6	0.057
	housekeeper vs. employed	2.40	−0.84, 5.6	0.150
	retirement/pension vs. employed	0.17	−0.64, 0.97	0.700

## Data Availability

The data presented in this study are available from the corresponding author upon reasonable request. Restrictions may apply due to ethical and privacy considerations.
